# Thermal management of micro-scale inorganic light-emittng diodes on an orthotropic substrate for biointegrated applications

**DOI:** 10.1038/s41598-017-06798-5

**Published:** 2017-07-26

**Authors:** Yuhang Li, Jin Chen, Yufeng Xing, Jizhou Song

**Affiliations:** 1 Institute of Solid Mechanics, Beihang University (BUAA), Beijing, 100191 China; 2 Key Laboratory of Soft Machines and Smart Devices of Zhejiang Province, Zhejiang University, Hangzhou, 310027 China; 3 State Key Laboratory of Digital Manufacturing Equipment and Technology, Huazhong University of Science and Technology, Wuhan, 430074 China; 4 Department of Engineering Mechanics and Soft Matter Research Center, Zhejiang University, Hangzhou, 310027 China

## Abstract

The orthotropic material with the in-plane thermal conductivity much larger than the off-plane one can control the heat flow direction. This feature provides unique benefits in thermal management of micro-scale inorganic light-emitting diodes (μ-ILEDs) device for biointegrated applications by helping the heat dissipation from μ-ILEDs along the in-plane directions to lower the μ-ILED temperature and prevent the heat dissipation to the tissue along the off-plane direction to ensure a low tissue temperature. Three-dimensional analytical models, accounting for the coupling between the Fourier heat conduction in the μ-ILED device and the Pennes bioheat transfer in the human skin, are established to investigate the thermal behaviors of μ-ILEDs on an orthotropic substrate integrated with the human skin. Both the operations of μ-ILEDs in a constant mode and pulsed mode are studied. The maximum temperature increases of μ-ILED and in the tissue are derived and their dependences on various parameters such as the thermal conductivities of the orthotropic substrate, substrate thickness, and loading parameters (e.g., duty cycle, pulse period) are investigated. These results pave the theoretical foundation for the thermal management of μ-ILED devices for biointegrated applications.

## Introduction

Recent advances in materials and mechanics^1–5^ enable high performance stretchable electronic and optoelectronic systems that are capable of wrapping on soft, curvilinear surfaces with applications in advanced biointegrated applications. Flexible and stretchable systems of microscale inorganic light-emitting diodes (μ-ILEDs) have attracted much attention due to their unique appealing optical advantages in would healing acceleration, photosensitive drug activation, wearable health/wellness monitoring and optogenetics^6–8^.

To achieve the mechanical compatibility with the biological tissue (e.g., human skin), the μ-ILEDs are integrated with a thin compliant substrate with the thickness on the order of ~100 μm or even smaller. The compliant substrate is usually made of a polymer with a low thermal conductivity on the order of 0.1 Wm^−1^K^−1^. Thermal management of μ-ILEDs is critically important for the design of μ-ILEDs for biointegrated applications because of the following reasons: (1) the low thermal conductivity of the substrate, which prevents the heat dissipation to the surrounding medium, may induce a high μ-ILED temperature rise to reduce its performance; (2) the small thickness of the substrate, which is benefit for heat dissipation to the tissue, may induce a high temperature rise at the substrate/tissue interface to cause tissue lesion or discomfort (even for a few degrees of temperature rise).

To reduce the adverse thermal effects of μ-ILEDs, many researchers have investigated the thermal behaviors of μ-ILEDs and developed design guidelines by optimizing the material, geometric and loading parameters^9–14^. Most of existing studies on thermal management of μ-ILEDs are for the system involving an isotropic substrate such that the heat dissipation in the substrate is uniform in the in-plane and off-plane (to the tissue) directions. It is hard to maintain the low temperature in μ-ILEDs and tissue simultaneously. The recent work on thermal metamaterials^15–17^ with the ability to control the heat flow direction could offer an appealing advantage to achieve the goal in thermal management. It is shown that the layered thermal metamaterials consisting of two layered materials with different thermal conductivities (e.g., metal and polymer) yields an orthotropic thermal behavior with the in-plane and off-plane thermal conductivities as

1 $${k}_{in-plane}=\frac{{k}_{1}+{k}_{2}}{2},{k}_{off-plane}=\frac{2{k}_{1}{k}_{2}}{{k}_{1}+{k}_{2}},$$

where *k*_1_ and *k*_2_ denote the thermal conductivities of two layered materials, respectively. For a large difference in *k*_1_ and *k*_2_, ${k}_{in-plane}\gg {k}_{off-plane}$, which indicates the heat dissipation along the in-plane directions is much larger than that along the off-plane direction. This orthotropic feature provides a unique benefit for thermal management of μ-ILEDs in biointegrated applications, which could help the heat dissipation from μ-ILEDs along the in-plane directions to lower the μ-ILED temperature increase^18^ and prevent the heat dissipation to the tissue along the off-plane direction to ensure a low tissue temperature increase.

The thermal behaviors of μ-ILEDs on an orthotropic substrate for biointegrated applications are rarely studied. In this paper, an analytical model, validated by finite element analysis (FEA), is established to investigate the thermal behaviors of μ-ILEDs on an orthotropic substrate integrated with human skin and to pave the theoretical foundation for the optimal design of μ-ILEDs to minimize the adverse thermal effects. The Pennes bioheat transfer equation, accounting for the effects of blood flow and metabolism, suffices for modeling the heat transfer in the skin and is given by,

2 $${k}_{skin}(\frac{{\partial }^{2}T}{\partial {x}^{2}}+\frac{{\partial }^{2}T}{\partial {y}^{2}}+\frac{{\partial }^{2}T}{\partial {z}^{2}})-{\varpi }_{b}{\rho }_{b}{c}_{b}(T-{T}_{b})+{q}_{met}={\rho }_{skin}{c}_{skin}\frac{\partial T}{\partial t},$$

where *k*_*skin*_ is the thermal conductivity of the skin, *ω*_*b*_ is the velocity of the blood flow, *ρ*_*b*_ is the density of the blood, *c*_*b*_ is the heat capacity of the blood, *T*_*b*_ is the temperature of the blood (which is usually taken as the body temperature), and *q*_*met*_ is the metabolic heat generation. Equation (2) becomes the Fourier heat conduction equation when the second and third terms on the left hand side are not included, which is applicable for modeling the heat transfer in μ-ILED device. The coupling between the Fourier heat conduction in μ-ILED device and the Pennes bioheat transfer in the human skin is accounted in the model, which can be easily extended to study μ-ILED system with different layouts and materials for various biointegrated applications.

## Thermal model under a constant power

**Figure 1:**
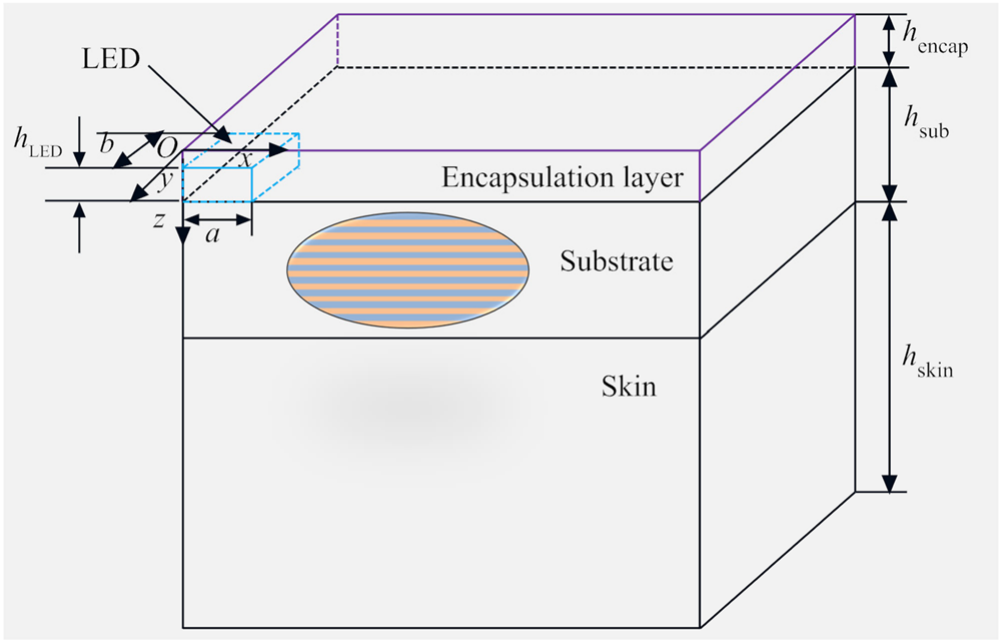
Schematic structure of the µ-ILED device on a layered orthotropic substrate integrated with human skin.

Figure 1 shows the typical layout of a μ-ILED system integrated with the human skin. Due to the symmetry of the structure, only one quarter geometry is illustrated. The μ-ILED system is composed of a μ-ILED encapsulated by an encapsulation layer on an orthotropic substrate consisting of two layered materials. The thicknesses of the μ-ILED, encapsulation layer, substrate and human skin are denoted by *h*_*LED*_, *h*_*encap*_, *h*_*sub*_, and *h*_*skin*_, respectively. The half-length and half-width of the μ-ILED are denoted by *a* and *b*, respectively. The coordinate system (*x*, *y*, *z*) is established with the origin located at the top surface as shown in Fig. 1. The μ-ILED is modeled as a planar heat source since the heat transfer mainly occurs through its top and bottom surface. For simplicity, the encapsulation layer is assumed to be an isotropic material. Under a constant power, the steady temperature *T*(*x*, *y*, *z*) satisfies

3 $$\begin{array}{cc}\frac{{\partial }^{2}T}{\partial {x}^{2}}+\frac{{\partial }^{2}T}{\partial {y}^{2}}+\frac{{\partial }^{2}T}{\partial {z}^{2}}=0 & 0\le z\le {h}_{encap}\\ {k}_{sub}^{x}\frac{{\partial }^{2}T}{\partial {x}^{2}}+{k}_{sub}^{y}\frac{{\partial }^{2}T}{\partial {y}^{2}}+{k}_{sub}^{z}\frac{{\partial }^{2}T}{\partial {z}^{2}}=0 & {h}_{encap}\le z\le {h}_{encap}+{h}_{sub}\\ {k}_{skin}(\frac{{\partial }^{2}T}{\partial {x}^{2}}+\frac{{\partial }^{2}T}{\partial {y}^{2}}+\frac{{\partial }^{2}T}{\partial {z}^{2}})-{\varpi }_{b}{\rho }_{b}{c}_{b}(T-{T}_{b})+{q}_{met}=0 & {h}_{encap}+{h}_{sub}\le z\le {h}_{encap}+{h}_{sub}+{h}_{skin},\end{array}$$

where the subscripts encap, sub, and skin represent the encapsulation, substrate and skin, respectively, and *k* is the thermal conductivity with the superscripts *x*, *y*, *z* denoting for the values along *x*, *y*, and *z* directions. The top surface has a natural convection boundary and the bottom surface has a constant core body temperature *T*_*body*_. The temperature of the blood is taken as the body temperature, i.e., *T*_*b*_ = *T*_*body*_. The temperature and heat flux are continuous across the skin/substrate interface. At the encapsulation/substrate interface, the temperature is continuous while the heat flux satisfies the heat source condition. These boundary and continuity conditions yield

4 $$\begin{array}{cc}{k}_{encap}\frac{\partial T}{\partial z}{|}_{z=0}=h(T-{T}_{\infty }) & z=0\\ {k}_{encap}\frac{\partial T}{\partial z}{|}_{z={h}_{encap}^{-}}-{k}_{sub}^{z}\frac{\partial T}{\partial z}{|}_{z={h}_{encap}^{+}}=\frac{{Q}_{0}}{4ab},T{|}_{z={h}_{encap}^{-}}=T{|}_{z={h}_{encap}^{+}} & z={h}_{encap},0\le x\le a,0\le y\le b\\ {k}_{encap}\frac{\partial T}{\partial z}{|}_{z={h}_{encap}^{-}}={k}_{sub}^{z}\frac{\partial T}{\partial z}{|}_{z={h}_{encap}^{+}},T{|}_{z={h}_{encap}^{-}}=T{|}_{z={h}_{encap}^{+}} & z={h}_{encap},x\ge a,y\ge b\\ {k}_{sub}^{z}\frac{\partial T}{\partial z}{|}_{z={({h}_{encap}+{h}_{sub})}^{-}}={k}_{skin}\frac{\partial T}{\partial z}{|}_{z={({h}_{encap}+{h}_{sub})}^{+}},T{|}_{z={({h}_{encap}+{h}_{sub})}^{-}}=T{|}_{z={({h}_{encap}+{h}_{sub})}^{+}} & z={h}_{encap}+{h}_{sub}\\ T{|}_{z={h}_{encap}+{h}_{sub}+{h}_{skin}}={T}_{body} & z={h}_{encap}+{h}_{sub}+{h}_{skin}\end{array}$$

with *h* as the coefficient of the natural convection and *T*_∞_ as the ambient temperature. The temperature *T*(*x*, *y*, *z*) can be solved by superposing the solutions of the following two problems:

(I) The steady temperature *T*_1_(*x*, *y*, *z*) when the power is not applied (i.e., the μ-ILED is not working) with the constant core body temperature at the bottom surface and the natural convection boundary at the top surface;
(II) The temperature increase *T*_2_(*x*, *y*, *z*) due to the applied power (i.e., the μ-ILED is working) with zero temperature at the bottom surface and the adiabatic condition at the top surface.

The problem (I) can be approximated by the one-dimensional heat transfer along the *z* direction since the size of μ-ILED is small comparing with other characteristic dimensions. Therefore, *T*_1_(*x*, *y*, *z*) ≈ *T*_1_(*z*), which satisfies

5 $$\begin{array}{cc}\frac{{d}^{2}{T}_{1}}{d{z}^{2}}=0 & 0\le z\le {h}_{encap}\\ \frac{{d}^{2}{T}_{1}}{d{z}^{2}}=0 & {h}_{encap}\le z\le {h}_{encap}+{h}_{sub}\\ {k}_{skin}\frac{{d}^{2}{T}_{1}}{d{z}^{2}}-{\varpi }_{b}{\rho }_{b}{c}_{b}({T}_{1}-{T}_{b})+{q}_{met}=0 & {h}_{encap}+{h}_{sub}\le z\le {h}_{encap}+{h}_{sub}+{h}_{skin},\end{array}$$

with boundary and continuity conditions

6 $$\begin{array}{c}{k}_{encap}\frac{d{T}_{1}}{dz}{|}_{z=0}=h({T}_{1}-{T}_{\infty })\\ {k}_{encap}\frac{d{T}_{1}}{dz}{|}_{z={h}_{encap}^{-}}={k}_{sub}^{z}\frac{d{T}_{1}}{dz}{|}_{z={h}_{encap}^{+}},{T}_{1}{|}_{z={h}_{encap}^{-}}={T}_{1}{|}_{z={h}_{encap}^{+}}\\ {k}_{sub}^{z}\frac{d{T}_{1}}{dz}{|}_{z={({h}_{encap}+{h}_{sub})}^{-}}={k}_{skin}\frac{d{T}_{1}}{dz}{|}_{z={({h}_{encap}+{h}_{sub})}^{+}},{T}_{1}{|}_{z={({h}_{encap}+{h}_{sub})}^{-}}={T}_{1}{|}_{z={({h}_{encap}+{h}_{sub})}^{+}}\\ {T}_{1}{|}_{z={h}_{encap}+{h}_{sub}+{h}_{skin}}={T}_{body}.\end{array}$$

*T*_1_ can then be obtained as

7 $$\begin{array}{cc}{T}_{1}^{encap}={A}_{1}z+{B}_{1}+{T}_{body} & 0\le z\le {h}_{encap}\\ {T}_{1}^{sub}={A}_{2}z+{B}_{2}+{T}_{body} & {h}_{encap}\le z\le {h}_{encap}+{h}_{sub}\\ {T}_{1}^{skin}={A}_{3}{e}^{\xi z}+{B}_{3}{e}^{-\xi z}+q+{T}_{body} & {h}_{encap}+{h}_{sub}\le z\le {h}_{encap}+{h}_{sub}+{h}_{skin}.\end{array}$$

where

8 $$\{\begin{array}{c}{A}_{1}\\ {B}_{1}\\ {A}_{2}\\ {B}_{2}\\ {A}_{3}\\ {B}_{3}\end{array}\}=\frac{1}{\varphi }\{\begin{array}{c}[q-{T}_{0}-\frac{2q}{\cosh ({h}_{skin}\xi )}]\frac{h}{{k}_{encap}}\\ {T}_{0}h\frac{{h}_{encap}}{{k}_{encap}}+{T}_{0}h\frac{{h}_{sub}}{{k}_{sub}^{z}}+q-\frac{2q}{\cosh ({h}_{skin}\xi )}+\frac{{T}_{0}h}{{k}_{skin}\xi }\,\tanh ({h}_{skin}\xi )\\ \left[q-{T}_{0}-\frac{2q}{\cosh ({h}_{skin}\xi )}\right]\frac{h}{{k}_{sub}^{z}}\\ (\frac{h{h}_{encap}}{{k}_{encap}}-\frac{h{t}_{encap}}{{k}_{sub}^{z}}+1)\frac{q[\cosh ({h}_{skin}\xi )-2]}{\cosh ({h}_{skin}\xi )}+\frac{{T}_{0}h({h}_{encap}+{h}_{sub})}{{k}_{sub}^{z}}+\frac{{T}_{0}h}{{k}_{skin}\xi }\,\tanh ({h}_{skin}\xi )\\ -(\frac{h{h}_{encap}}{{k}_{encap}}+\frac{h{h}_{sub}}{{k}_{sub}^{z}}+1)\frac{q{e}^{-({h}_{encap}+{h}_{sub})\xi }}{\cosh ({h}_{skin}\xi )}-\frac{h{e}^{-({h}_{encap}+{h}_{sub})\xi }}{{k}_{skin}\xi \,\cosh ({h}_{skin}\xi )}[q+({T}_{0}-q){e}^{-{h}_{skin}\xi }]\\ -(\frac{h{h}_{encap}}{{k}_{encap}}+\frac{h{h}_{sub}}{{k}_{sub}^{z}}+1)\frac{q{e}^{({h}_{encap}+{h}_{sub})\xi }}{\cosh ({h}_{skin}\xi )}+\frac{h{e}^{({h}_{encap}+{h}_{sub})\xi }}{{k}_{skin}\xi \,\cosh ({h}_{skin}\xi )}[q+({T}_{0}-q){e}^{{h}_{skin}\xi }]\end{array}\}.$$

with $\xi =\sqrt{{\omega }_{b}{\rho }_{b}{c}_{b}/{k}_{skin}}$, *q* = *q*_*met*_/(*ω*_*b*_ *ρ*_*b*_ *c*_*b*_), *T*_0_ = *T*_∞_ − *T*_*body*_ and

9 $$\varphi =(\frac{h{h}_{encap}}{{k}_{encap}}+\frac{h{h}_{sub}}{{k}_{sub}^{z}}+1)+\frac{h}{{k}_{skin}\xi }\,\tanh ({h}_{skin}\xi ).$$

The temperatures at the skin/substrate interface and in μ-ILED due to the heating of the body are then given by

10 $${T}_{1}^{skin/sub}={A}_{2}({h}_{encap}+{h}_{sub})+{B}_{2}+{T}_{body}$$

and

11 $${T}_{1}^{LED}={A}_{2}{h}_{encap}+{B}_{2}+{T}_{body}.$$

The solution *T*_2_(*x*, *y*, *z*) for the problem (II) satisfies

12 $$\begin{array}{cc}\frac{{\partial }^{2}{T}_{2}}{\partial {x}^{2}}+\frac{{\partial }^{2}{T}_{2}}{\partial {y}^{2}}+\frac{{\partial }^{2}{T}_{2}}{\partial {z}^{2}}=0 & 0\le z\le {h}_{encap}\\ {k}_{sub}^{x}\frac{{\partial }^{2}{T}_{2}}{\partial {x}^{2}}+{k}_{sub}^{y}\frac{{\partial }^{2}{T}_{2}}{\partial {y}^{2}}+{k}_{sub}^{z}\frac{{\partial }^{2}{T}_{2}}{\partial {z}^{2}}=0 & {h}_{encap}\le z\le {h}_{encap}+{h}_{sub}\\ {k}_{skin}(\frac{{\partial }^{2}{T}_{2}}{\partial {x}^{2}}+\frac{{\partial }^{2}{T}_{2}}{\partial {y}^{2}}+\frac{{\partial }^{2}{T}_{2}}{\partial {z}^{2}})-{\varpi }_{b}{\rho }_{b}{c}_{b}{T}_{2}=0 & {h}_{encap}+{h}_{sub}\le z\le {h}_{encap}+{h}_{sub}+{h}_{skin},\end{array}$$

with boundary and continuity conditions

13 $$\begin{array}{cc}{k}_{encap}\frac{\partial {T}_{2}}{\partial z}{|}_{z=0}=0 & z=0\\ {k}_{encap}\frac{\partial {T}_{2}}{\partial z}{|}_{z={h}_{encap}^{-}}-{k}_{sub}^{z}\frac{\partial {T}_{2}}{\partial z}{|}_{z={h}_{encap}^{+}}=\frac{{Q}_{0}}{4ab},{T}_{2}{|}_{z={h}_{encap}^{-}}={T}_{2}{|}_{z={h}_{encap}^{+}} & z={h}_{encap},0\le x\le a,0\le y\le b\\ {k}_{encap}\frac{\partial {T}_{2}}{\partial z}{|}_{z={h}_{encap}^{-}}={k}_{sub}^{z}\frac{\partial {T}_{2}}{\partial z}{|}_{z={h}_{encap}^{+}},{T}_{2}{|}_{z={h}_{encap}^{-}}={T}_{2}{|}_{z={h}_{encap}^{+}} & z={h}_{encap},x\ge a,y\ge b\\ {k}_{sub}^{z}\frac{\partial {T}_{2}}{\partial z}{|}_{z={({h}_{encap}+{h}_{sub})}^{-}}={k}_{skin}\frac{\partial {T}_{2}}{\partial z}{|}_{z={({h}_{encap}+{h}_{sub})}^{+}},{T}_{2}{|}_{z={({h}_{encap}+{h}_{sub})}^{-}}={T}_{2}{|}_{z={({h}_{encap}+{h}_{sub})}^{+}} & z={h}_{encap}+{h}_{sub}\\ {T}_{2}{|}_{z={h}_{encap}+{h}_{sub}+{h}_{skin}}=0 & z={h}_{encap}+{h}_{sub}+{h}_{skin}\end{array}$$

The Fourier Cosine transform ${\hat{T}}_{2}(\alpha ,\beta ,z)={\int }_{0}^{\infty }{\int }_{0}^{\infty }{T}_{2}(x,y,z)\cos (\alpha x)\cos (\beta y)dxdy$ is applied to Eqs (12) and (13), and yields

14 $$\begin{array}{cc}\frac{{\partial }^{2}{\hat{T}}_{2}}{\partial {z}^{2}}-{\xi }_{1}^{2}{\hat{T}}_{2}=0 & 0\le z\le {h}_{encap}\\ \frac{{\partial }^{2}{\hat{T}}_{2}}{\partial {z}^{2}}-{\xi }_{2}^{2}{\hat{T}}_{2}=0 & {h}_{encap}\le z\le {h}_{encap}+{h}_{sub}\\ \frac{{\partial }^{2}{\hat{T}}_{2}}{\partial {z}^{2}}-{\xi }_{3}^{2}{\hat{T}}_{2}=0 & {h}_{encap}+{h}_{sub}\le z\le {h}_{encap}+{h}_{sub}+{h}_{skin}\end{array}$$

and

15 $$\begin{array}{c}{k}_{encap}\frac{d{\hat{T}}_{2}}{dz}{|}_{z=0}=0\\ {k}_{encap}\frac{d{\hat{T}}_{2}}{dz}{|}_{z={h}_{encap}^{-}}-{k}_{sub}^{z}\frac{d{\hat{T}}_{2}}{dz}{|}_{z={h}_{encap}^{+}}=\frac{{Q}_{0}\,\sin (\alpha a)\sin (\beta b)}{4ab\alpha \beta },{\hat{T}}_{2}{|}_{z={h}_{encap}^{-}}={\hat{T}}_{2}{|}_{z={h}_{encap}^{+}}\\ {k}_{sub}^{z}\frac{d{\hat{T}}_{2}}{dz}{|}_{z={({h}_{encap}+{h}_{sub})}^{-}}={k}_{skin}\frac{d{\hat{T}}_{2}}{dz}{|}_{z={({h}_{encap}+{h}_{sub})}^{+}},{\hat{T}}_{2}{|}_{z={({h}_{encap}+{h}_{sub})}^{-}}={\hat{T}}_{2}{|}_{z={({h}_{encap}+{h}_{sub})}^{+}}\\ {\hat{T}}_{2}{|}_{z={h}_{encap}+{h}_{sub}+{h}_{skin}}=0,\end{array}$$

with *η* ^2^ = *ϖ*_*b*_ *ρ*_*b*_ *c*_*b*_/*k*_*skin*_, ${\xi }_{1}=\sqrt{{\alpha }^{2}+{\beta }^{2}}$, ${\xi }_{2}=\sqrt{({k}_{sub}^{x}{\alpha }^{2}+{k}_{sub}^{y}{\beta }^{2})/{k}_{sub}^{z}}$, and ${\xi }_{3}=\sqrt{{\alpha }^{2}+{\beta }^{2}+{\eta }^{2}}$. Equations (14) and (15) have the following solutions

16 $$\begin{array}{cc}{\hat{T}}_{2}^{encap}={\hat{A}}_{1}\,\sinh ({\xi }_{1}z)+{\hat{B}}_{1}\,\cosh ({\xi }_{1}z) & 0\le z\le {h}_{encap}\\ {\hat{T}}_{2}^{sub}={\hat{A}}_{2}\,\sinh ({\xi }_{2}z)+{\hat{B}}_{2}\,\cosh ({\xi }_{2}z) & {h}_{encap}\le z\le {h}_{encap}+{h}_{sub}\\ {\hat{T}}_{2}^{skin}={\hat{A}}_{3}\,\sinh ({\xi }_{3}(z-({h}_{encap}+{h}_{sub}+{h}_{skin})) & {h}_{encap}+{h}_{sub}\le z\le {h}_{encap}+{h}_{sub}+{h}_{skin},\end{array}$$

where the coefficients $\hat{A}$ and $\hat{B}$ are obtained as

17 $$\{\begin{array}{c}{\hat{A}}_{1}\\ {\hat{B}}_{1}\\ {\hat{A}}_{2}\\ {\hat{B}}_{2}\\ {\hat{A}}_{3}\end{array}\}=\frac{1}{\delta }\{\begin{array}{c}0\\ \left[{k}_{sub}^{z}{\xi }_{2}\,\tanh ({\xi }_{3}{h}_{skin})+{k}_{skin}{\xi }_{3}\,\tanh ({\xi }_{2}{h}_{sub})\right]\frac{{\xi }_{encap}}{\cosh ({\xi }_{1}{h}_{encap})}\\ -\frac{{k}_{sub}^{z}{\xi }_{1}{\xi }_{2}\,\sinh [{\xi }_{2}({h}_{encap}+{h}_{sub})]\tanh ({\xi }_{3}{h}_{skin})+{k}_{skin}{\xi }_{1}{\xi }_{3}\,\cosh [{\xi }_{2}({h}_{encap}+{h}_{sub})]}{\cosh ({\xi }_{2}{h}_{sub})}\\ \frac{{k}_{sub}^{z}{\xi }_{1}{\xi }_{2}\,\cosh [{\xi }_{2}({h}_{encap}+{h}_{sub})]\tanh ({\xi }_{3}{h}_{skin})+{k}_{skin}{\xi }_{1}{\xi }_{3}\,\sinh [{\xi }_{2}({h}_{encap}+{h}_{sub})]}{\cosh ({\xi }_{2}{h}_{sub})}\\ -\frac{{k}_{sub}^{z}{\xi }_{1}{\xi }_{2}}{\cosh ({\xi }_{2}{h}_{sub})\cosh ({\xi }_{3}{h}_{skin})}\end{array}\}.$$

with *δ* as

18 $$\delta =[\begin{array}{c}{k}_{encap}{k}_{sub}^{z}{\xi }_{1}{\xi }_{2}\,\tanh ({\xi }_{1}{h}_{encap})\tanh ({\xi }_{3}{h}_{skin})\\ +{k}_{encap}{k}_{skin}{\xi }_{1}{\xi }_{3}\,\tanh ({\xi }_{1}{h}_{encap})\tanh ({\xi }_{2}{h}_{sub})\\ +{k}_{sub}^{z2}{\xi }_{2}^{2}\,\tanh ({\xi }_{2}{h}_{sub})\tanh ({\xi }_{3}{h}_{skin})+{k}_{sub}^{z}{k}_{skin}{\xi }_{2}{\xi }_{3}\end{array}]\frac{\alpha \beta ab{\xi }_{1}}{{Q}_{0}\,\sin (\alpha a)\sin (\beta b)}.$$

The inverse Cosine transform ${T}_{2}(x,y,z)=\frac{4}{{\pi }^{2}}{\int }_{0}^{\infty }{\int }_{0}^{\infty }{\hat{T}}_{2}(\alpha ,\beta ,z)\cos (\alpha x)\cos (\beta y)d\alpha d\beta$ gives the temperature rise due to the applied power *Q*_0_ in the encapsulation, substrate and skin. For example, the temperature rise in the substrate is given by

19 $${T}_{2}^{sub}(x,y,z)=\frac{4}{{\pi }^{2}}{\int }_{0}^{\infty }{\int }_{0}^{\infty }[{\hat{A}}_{2}\,\sinh ({\xi }_{2}z)+{\hat{B}}_{2}\,\cosh ({\xi }_{2}z)]\cos (\alpha x)\cos (\beta y)d\alpha d\beta .$$

The maximum temperature increase in the skin due to the applied power occurs at the point *A* (0, 0, *h*_*encap*_ + *h*_*sub*_) on the skin/substrate interface and is given by

20 $$\begin{array}{rcl}{({T}_{2}^{skin})}_{\max } & = & {T}_{2}^{sub}(0,0,{h}_{encap}+{h}_{sub})\\  & = & \frac{4}{{\pi }^{2}}{\int }_{0}^{\infty }{\int }_{0}^{\infty }\{{\hat{A}}_{2}\,\sinh \,[{\xi }_{2}({h}_{encap}+{h}_{sub})]+{\hat{B}}_{2}\,\cosh \,[{\xi }_{2}({h}_{encap}+{h}_{sub})]\}d\alpha d\beta .\end{array}$$

The temperature rise of μ-ILED can be obtained by averaging the temperature rise over the whole surface of μ-ILED, i.e.,

21 $${T}_{2}^{LED}={\int }_{0}^{b}{\int }_{0}^{a}\frac{{T}_{2}^{sub}(x,y,{h}_{encap})}{ab+\frac{(a+b){h}_{LED}}{2}}dxdy.$$

The superposition of the problem (I) and (II) then gives the temperature distributions under a constant power. The maximum temperature in the skin, which occurs at the skin/substrate interface, and the μ-ILED temperature are obtained as

22 $${({T}^{skin})}_{\max }={T}_{1}^{skin/sub}+{({T}_{2}^{skin})}_{\max },$$

and

23 $${T}^{LED}={T}_{1}^{LED}+{T}_{2}^{LED},$$

respectively. The above two equations serve as the theoretical basis in thermal management for μ-ILEDs in biointegrated applications under a constant power.

## Thermal analysis under a pulsed power

The μ-ILEDs in pulsed operation can provide an effective strategy in thermal management^10–12^ and more benefits in biointegrated applications such as optogenetics^7^, where a pulsed mode operation is required. Under a pulsed power, the temperature in the system increases a fluctuating way and then reaches saturation within a constant band. Let *θ*(*x*, *y*, *z*, *t*) denote the saturation temperature under a pulsed power *Q*(*t*), which satisfies the transient heat conduction equations

24 $$\begin{array}{cc}{\lambda }_{encap}(\frac{{\partial }^{2}\theta }{\partial {x}^{2}}+\frac{{\partial }^{2}\theta }{\partial {y}^{2}}+\frac{{\partial }^{2}\theta }{\partial {z}^{2}})-\frac{\partial \theta }{\partial t}=0 & 0\le z\le {h}_{encap}\\ {\lambda }_{sub}^{x}\frac{{\partial }^{2}\theta }{\partial {x}^{2}}+{\lambda }_{sub}^{y}\frac{{\partial }^{2}\theta }{\partial {y}^{2}}+{\lambda }_{sub}^{z}\frac{{\partial }^{2}\theta }{\partial {z}^{2}}-\frac{\partial \theta }{\partial t}=0 & {h}_{encap}\le z\le {h}_{encap}+{h}_{sub}\\ {\lambda }_{skin}(\frac{{\partial }^{2}\theta }{\partial {x}^{2}}+\frac{{\partial }^{2}\theta }{\partial {y}^{2}}+\frac{{\partial }^{2}\theta }{\partial {z}^{2}})-\frac{\partial \theta }{\partial t}-\frac{{\varpi }_{b}{\rho }_{b}{c}_{b}}{{\rho }_{skin}{c}_{skin}}(\theta -{T}_{b})+\frac{{q}_{met}}{{\rho }_{skin}{c}_{skin}}=0 & {h}_{encap}+{h}_{sub}\le z\le {h}_{encap}+{h}_{sub}+{h}_{skin},\end{array}$$

with the boundary and continuity conditions same as Eq. (4) except that a constant power *Q*_0_ is replaced by the pulsed power *Q*(*t*). Here *ρ* and *c* are the mass density and heat capacity, respectively, *λ* = *k*/(*ρc*) is the thermal diffusivity. Similar to the case under a constant power, the temperature *θ*(*x*, *y*, *z*, *t*) can also be solved by superposing the solutions of the two problems:

(I) The steady temperature *θ*_1_(*x*, *y*, *z*) when the power is not applied (i.e., the μ-ILED is not working) with the constant core body temperature at the bottom surface and the natural convection boundary at the top surface;
(II) The temperature increase *θ*_2_(*x*, *y*, *z*, *t*) due to the applied power (i.e., the μ-ILED is working) with zero temperature at the bottom surface and the adiabatic condition at the top surface.

The steady temperature *θ*_1_ is same as *T*_1_ and therefore, we will focus on solving *θ*_2_(*x*, *y*, *z*, *t*) below, which satisfies

25 $$\begin{array}{cc}{\lambda }_{encap}(\frac{{\partial }^{2}{\theta }_{2}}{\partial {x}^{2}}+\frac{{\partial }^{2}{\theta }_{2}}{\partial {y}^{2}}+\frac{{\partial }^{2}{\theta }_{2}}{\partial {z}^{2}})-\frac{\partial {\theta }_{2}}{\partial t}=0 & 0\le z\le {h}_{encap}\\ {\lambda }_{sub}^{x}\frac{{\partial }^{2}{\theta }_{2}}{\partial {x}^{2}}+{\lambda }_{sub}^{y}\frac{{\partial }^{2}{\theta }_{2}}{\partial {y}^{2}}+{\lambda }_{sub}^{z}\frac{{\partial }^{2}{\theta }_{2}}{\partial {z}^{2}}-\frac{\partial {\theta }_{2}}{\partial t}=0 & {h}_{encap}\le z\le {h}_{encap}+{h}_{sub}\\ {\lambda }_{skin}(\frac{{\partial }^{2}{\theta }_{2}}{\partial {x}^{2}}+\frac{{\partial }^{2}{\theta }_{2}}{\partial {y}^{2}}+\frac{{\partial }^{2}{\theta }_{2}}{\partial {z}^{2}})-\frac{\partial {\theta }_{2}}{\partial t}-\frac{{\varpi }_{b}{\rho }_{b}{c}_{b}}{{\rho }_{skin}{c}_{skin}}{\theta }_{2}=0 & {h}_{encap}+{h}_{sub}\le z\le {h}_{encap}+{h}_{sub}+{h}_{skin},\end{array}$$

with the boundary and continuity conditions same as Eq. (13) except that a constant power *Q*_0_ is replaced by the pulsed power *Q*(*t*).

The pulsed power can be expressed via its Fourier series by

26 $$Q(t)={Q}_{0}\{\begin{array}{cc}1 & 0 < t\le \tau \\ 0 & \tau  < t\le {t}_{0}\end{array}={Q}_{0}[{a}_{0}+\sum _{n=1}^{\infty }({a}_{n}\,\cos \,n\omega t+{b}_{n}\,\sin \,n\omega t)],$$

where *τ* is the pulse duration time, *t*_0_ is the pulse period, *Q*_0_ is the power amplitude, *a*_0_ = *D* = *τ*/*t*_0_, *ω* = 2*π*/*t*_0_, ${a}_{n}=\sin (2n\pi D)/(n\pi )$, and ${b}_{n}=[1-\,\cos (2n\pi D)]/(n\pi )$. The superposition of the solution for each sinusoidal power ${Q}_{0}\,\sin \,n\omega t$ (or ${Q}_{0}\,\cos \,n\omega t$) gives *θ*_2_(*x*, *y*, *z*, *t*).

For a power of *Q*_0_ *e* ^*nωt*⋅*i*^, the temperature takes the form of *ϕ*(*x*, *y*, *z*; *nω*)*e* ^*nωt*⋅*i*^ with the real part $|\phi (x,y,z;n\omega )|\cos (n\omega t+{\gamma }_{n})$ as the solution for the power ${Q}_{0}\,\cos (n\omega t)$ and the imaginary part $|\phi (x,y,z;n\omega )|\sin (n\omega t+{\gamma }_{n})$ as the solution for the power ${Q}_{0}\,\sin (n\omega t)$. Here *γ*_*n*_ is the phase angle of *ϕ*(*x*, *y*, *z*; *nω*). *θ*_2_(*x*, *y*, *z*, *t*) is then given by

27 $${\theta }_{2}(x,y,z,t)=D\phi (0)+\sum _{n=1}^{\infty }|\phi (n\omega )|[\begin{array}{c}\frac{\sin (2n\pi D)}{n\pi }\,\cos (n\omega t+{\gamma }_{n})\\ +\frac{1-\,\cos (2n\pi D)}{n\pi }\,\sin (n\omega t+{\gamma }_{n})\end{array}].$$

The substitution of *ϕ*(*x*, *y*, *z*; *nω*)*e* ^*nωt*⋅*i*^ into Eq. (25) yields

28 $$\begin{array}{cc}{\lambda }_{encap}(\frac{{\partial }^{2}\phi }{\partial {x}^{2}}+\frac{{\partial }^{2}\phi }{\partial {y}^{2}}+\frac{{\partial }^{2}\phi }{\partial {z}^{2}})-n\omega i\frac{\partial \phi }{\partial t}=0 & 0\le z\le {h}_{encap}\\ {\lambda }_{sub}^{x}\frac{{\partial }^{2}\phi }{\partial {x}^{2}}+{\lambda }_{sub}^{y}\frac{{\partial }^{2}\phi }{\partial {y}^{2}}+{\lambda }_{sub}^{z}\frac{{\partial }^{2}\phi }{\partial {z}^{2}}-n\omega i\frac{\partial \phi }{\partial t}=0 & {h}_{encap}\le z\le {h}_{encap}+{h}_{sub}\\ {\lambda }_{skin}(\frac{{\partial }^{2}\phi }{\partial {x}^{2}}+\frac{{\partial }^{2}\phi }{\partial {y}^{2}}+\frac{{\partial }^{2}\phi }{\partial {z}^{2}})-n\omega i\frac{\partial \phi }{\partial t}-\frac{{\varpi }_{b}{\rho }_{b}{c}_{b}}{{\rho }_{skin}{c}_{skin}}\phi =0 & {h}_{encap}+{h}_{sub}\le z\le {h}_{encap}+{h}_{sub}+{h}_{skin}.\end{array}$$

The boundary and continuity conditions for *ϕ* are same as Eq. (13). After the Fourier Cosine transform, Equation (28) becomes

29 $$\begin{array}{cc}\frac{{\partial }^{2}\hat{\phi }}{\partial {z}^{2}}-{\zeta }_{1}^{2}\hat{\phi }=0 & 0\le z\le {h}_{encap}\\ \frac{{\partial }^{2}\hat{\phi }}{\partial {z}^{2}}-{\zeta }_{2}^{2}\hat{\phi }=0 & {h}_{encap}\le z\le {h}_{encap}+{h}_{sub}\\ \frac{{\partial }^{2}\hat{\phi }}{\partial {z}^{2}}-{\zeta }_{3}^{2}\hat{\phi }=0 & {h}_{encap}+{h}_{sub}\le z\le {h}_{encap}+{h}_{sub}+{h}_{skin}\end{array}$$

with ${\zeta }_{1}=\sqrt{{\alpha }^{2}+{\beta }^{2}+n\omega i/{\lambda }_{encap}}$, ${\zeta }_{2}=\sqrt{({\lambda }_{sub}^{x}{\alpha }^{2}+{\lambda }_{sub}^{y}{\beta }^{2}+n\omega i)/{\lambda }_{sub}^{z}}$, and ${\zeta }_{3}=\sqrt{{\alpha }^{2}+{\beta }^{2}+n\omega i/{\lambda }_{skin}+{\varpi }_{b}{\rho }_{b}{c}_{b}/{k}_{skin}}$. It is noted that the governing equations, boundary and continuity conditions for $\hat{\phi }$ are same as those for ${\hat{T}}_{2}$ except that *ξ* is replaced by *ζ*. Therefore, the substitution of *ξ* by *ζ* in ${\hat{T}}_{2}$ gives $\hat{\phi }$. The inverse Cosine transform of $\hat{\phi }$ then gives *ϕ*. For example, the solution of *ϕ* in the substrate layer is given by

30 $${\phi }^{sub}(x,y,z;n\omega )=\frac{4}{{\pi }^{2}}{\int }_{0}^{\infty }{\int }_{0}^{\infty }[{\hat{E}}_{2}\,\sinh ({\zeta }_{2}z)+{\hat{F}}_{2}\,\cosh ({\zeta }_{2}z)]\cos (\alpha x)\cos (\beta y)d\alpha d\beta ,$$

where

31 $$\{\begin{array}{c}{\hat{E}}_{2}\\ {\hat{F}}_{2}\end{array}\}=\frac{1}{G}\{\begin{array}{c}-{k}_{sub}^{z}{\zeta }_{2}\,\sinh [{\zeta }_{2}({h}_{encap}+{h}_{sub})]\tanh ({\zeta }_{3}{h}_{skin})-{k}_{skin}{\zeta }_{3}\,\cosh [{\zeta }_{2}({h}_{encap}+{h}_{sub})]\\ {k}_{sub}^{z}{\zeta }_{2}\,\cosh [{\zeta }_{2}({h}_{encap}+{h}_{sub})]\tanh ({\zeta }_{3}{h}_{skin})+{k}_{skin}{\zeta }_{3}\,\sinh [{\zeta }_{2}({h}_{encap}+{h}_{sub})]\end{array}\}$$

with *G* as

32 $$G=[\begin{array}{c}{k}_{encap}{k}_{sub}^{z}{\zeta }_{1}{\zeta }_{2}\,\tanh ({\zeta }_{1}{h}_{encap})\tanh ({\zeta }_{3}{h}_{skin})\\ +{k}_{encap}{k}_{skin}{\zeta }_{1}{\zeta }_{3}\,\tanh ({\zeta }_{1}{h}_{encap})\tanh ({\zeta }_{2}{h}_{sub})\\ +{k}_{sub}^{z2}{\zeta }_{2}^{2}\,\tanh ({\zeta }_{2}{h}_{sub})\tanh ({\zeta }_{3}{h}_{skin})+{k}_{sub}^{z}{k}_{skin}{\zeta }_{2}{\zeta }_{3}\end{array}]\frac{\alpha \beta ab\,\cosh ({\zeta }_{2}{h}_{sub})}{{Q}_{0}\,\sin (\alpha a)\sin (\beta b)}.$$

The temperature rise due to the power *Q*_0_ *e* ^*nωt*⋅*i*^ at point *A* (0, 0, *h*_*encap*_ + *h*_*sub*_) on the skin/substrate interface and μ-ILED are obtained by

33 $${\phi }_{A}^{skin/sub}(n\omega )=\frac{4}{{\pi }^{2}}{\int }_{0}^{\infty }{\int }_{0}^{\infty }\{{\hat{E}}_{2}\,\sinh \,[{\zeta }_{2}({h}_{encap}+{h}_{sub})]+{\hat{F}}_{2}\,\cosh \,[{\zeta }_{2}({h}_{encap}+{h}_{sub})]\}d\alpha d\beta $$

and

34 $${\phi }^{LED}(n\omega )={\int }_{0}^{b}{\int }_{0}^{a}\frac{{\phi }^{sub}(x,y,{h}_{encap})}{ab+\frac{(a+b){h}_{LED}}{2}}{\rm{d}}xdy,$$

respectively. The temperature rise under a pulsed a power in problem (II) at point *A* on the skin/substrate interface and μ-ILED are then given by

35 $${({\theta }_{2}^{skin/sub})}_{A}(t)=D{\phi }_{A}^{skin/sub}(0)+\sum _{n=1}^{\infty }|{\phi }_{A}^{skin/sub}(n\omega )|[\begin{array}{c}\frac{\sin (2n\pi D)}{n\pi }\,\cos (n\omega t+{\gamma }_{n})\\ +\frac{1-\,\cos (2n\pi D)}{n\pi }\,\sin (n\omega t+{\gamma }_{n})\end{array}],$$

and

36 $${\theta }_{2}^{LED}(t)=D{\phi }^{LED}(0)+\sum _{n=1}^{\infty }|{\phi }^{LED}(n\omega )|[\begin{array}{c}\frac{\sin (2n\pi D)}{n\pi }\,\cos (n\omega t+{\gamma }_{n})\\ +\frac{1-\,\cos (2n\pi D)}{n\pi }\,\sin (n\omega t+{\gamma }_{n})\end{array}].$$

Finally, the maximum temperature rise of the skin and μ-ILED under a pulsed a power then given by

37 $${({\theta }^{skin})}_{\max }={T}_{1}^{skin/sub}+{({\theta }_{2}^{skin/sub})}_{A}^{\max },$$

and

38 $${({\theta }^{LED})}_{\max }={T}_{1}^{LED}+{({\theta }_{2}^{LED})}_{\max },$$

which pave the theoretical foundation in thermal management for the design of μ-ILEDs in biointegrated applications under a pulsed power.

## Results and Discussion

We take SU8 as the encapsulation layer from experiments with the thermal conductivity, specific heat capacity and density as *k*_*encap*_ = 0.2 W/m/K, *c*_*encap*_ = 1200 J/kg/K, and *ρ*_*encap*_ = 1090 kg/m^3^, respectively^19^. The thermal conductivity along the *z* direction, specific heat capacity and density of the substrate are ${k}_{sub}^{z}=0.17\,W/m/K$, *c*_*sub*_ = 1460 J/kg/K, and *ρ*_*sub*_ = 970 kg/m^3^, which correspond to those of PDMS^20^. The thermal conductivity, specific heat capacity and density of the skin are *k*_*skin*_ = 0.37 W/m/K, *c*_*skin*_ = 2846 J/kg/K, and *ρ*_*skin*_ = 1000 kg/m^3^ ^21^. The thicknesses of the encapsulation layer and μ-ILED are set as *h*_*encap*_ = 7 μm and *h*_*LED*_ = 6.5 μm from experiments^19^. Unless otherwise specified, the substrate thickness is *h*_*sub*_ = 200 μm, the in-plane thermal conductivity of the substrate is ${k}_{sub}^{x}={k}_{sub}^{y}=1.7\,W/m/K$, and the size of μ-ILED is 2*a* × 2*b* = 100 μm × 100 μm. The parameters for the skin are *h*_*skin*_ = 4 mm for the thickness, *ω*_*b*_ = 0.05 ml Blood/ml tissue/s for the blood flow velocity, *ρ*_*b*_ = 1000 kg/m^3^ for the blood density, *c*_*b*_ = 4218 J/kg/K for the specific heat capacity of the blood, and *q*_*met*_ = 368.1 W/m^3^ for the metabolic heat generation^21^.

A three-dimensional finite element model is established in ABAQUS to validate the analytical models. The in-plane dimension of the encapsulation, substrate and skin is 12 mm × 12 mm. The side surfaces are all adiabatic boundaries. The top surface of the encapsulation layer has a natural convection boundary with the coefficient of heat convection as *h* = 25 W/m^2^/K. The bottom surface of the skin is *T*_*body*_ = 37°C, which is equal to the core body temperature. The ambient temperature is *T*_∞_ = 25°C. The continuous element DC3D8 is used to discretize the geometry. When a power is applied, the μ-ILED is modeled as a volume heat source. The applied power is set as *Q*_0_ = 10 mW in all calculations.

**Figure 2:**
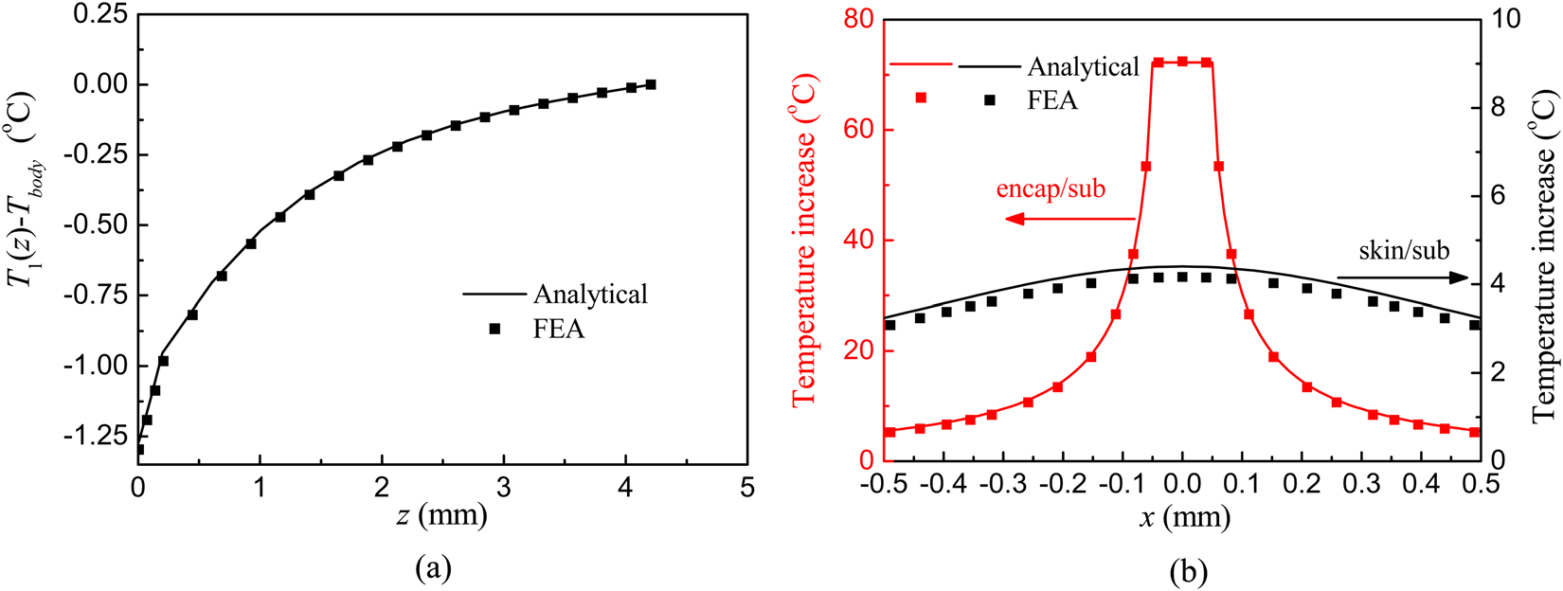
(**a**) The distribution of the steady temperature increase from the core body temperature when μ-ILED is not working. (**b**) The distribution of the temperature increase due to the applied power of 10 mW at the encapsulation/substrate and skin/substrate interfaces.

Figure 2(a) shows the distribution of the steady temperature increase from the core body temperature *T*_1_(*z*) − *T*_*body*_ when the μ-ILED is not working. The good agreement between the analytical prediction and FEA validates the analytical model. It can be found that the temperature increase decays exponentially in the skin due to the Pennes bioheat transfer and then decays linearly in the substrate and encapsulation layers due to the Fourier heat conduction. The steady temperature increase of μ-ILED is −1.3 °C while the temperature increase at the skin/substrate is −1 °C. When the power *Q*_0_ = 10 mW is applied, the distributions of the temperature increase *T*_2_(*x*, 0, *z*) at the encapsulation/substrate (*z* = *h*_*encap*_) and skin/substrate (*z* = *h*_*encap*_ + *h*_*sub*_) interfaces along *x* direction are shown in Fig. 2(b). The analytical predictions agree well with FEA. At the encapsulation/substrate interface, the maximum temperature occurs within the region of μ-ILED (−50 μm ≤ *x* ≤ 50 μm) and then decays quickly as the distance to μ-ILED increases. The temperature increase at the skin/substrate interface becomes more flat due to the heat sink effect of the skin.

**Figure 3:**
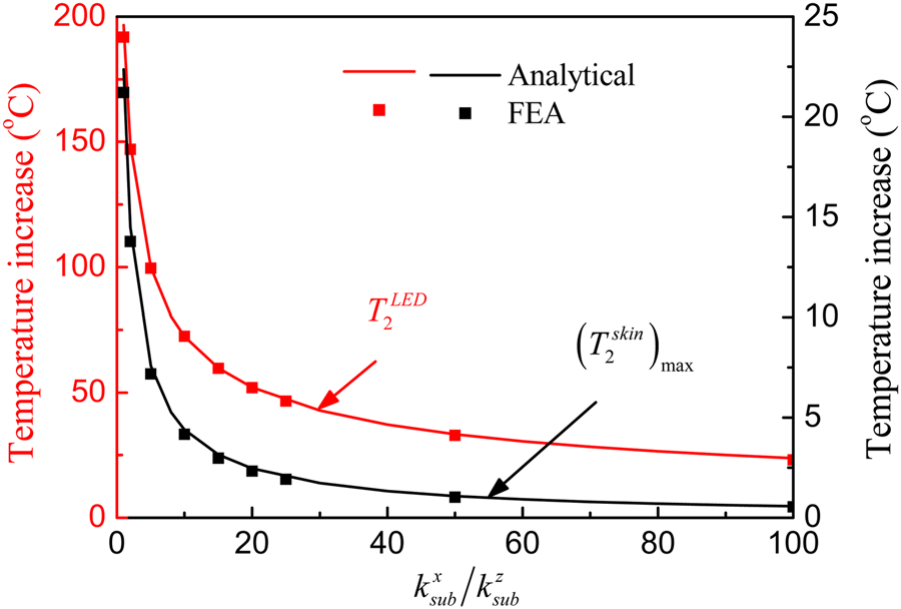
Effects of the orthotropic substrate on the μ-ILED temperature increase and the maximum skin temperature increase under a constant power of 10 mW.

The influences of the orthotropic substrate on the μ-ILED temperature increase ${T}_{2}^{LED}$ and the maximum temperature increase ${({T}_{2}^{skin})}_{\max }$ in the skin under a constant power of 10 mW are shown in Fig. 3. The thermal conductivity of the substrate along the *z* direction is fixed as ${k}_{sub}^{z}=0.17\,W/m/K$. The maximum temperature increase in the skin is much lower than the μ-ILED temperature increase due to the substrate’s thermal insulation effect. The increase of the in-plane thermal conductivity (${k}_{sub}^{x}$ and ${k}_{sub}^{y}$) of the substrate increases more heat to dissipate along the in-plane directions, and reduces both the μ-ILED temperature increase and the maximum temperature increase in the skin. As the thermal conductivity ratio ${k}_{sub}^{x}/{k}_{sub}^{z}$ increases from 1, corresponding to an isotropic substrate, to 20, the μ-ILED temperature increase drops 73.4% from 196.6 °C to 52.1 °C and the maximum temperature increase in the skin drops 88.8% from 22.4 °C to 2.5 °C. As the thermal conductivity ratio ${k}_{sub}^{x}/{k}_{sub}^{z}$ further increases, both ${T}_{2}^{LED}$ and ${({T}_{2}^{skin})}_{\max }$ drop slowly.

**Figure 4:**
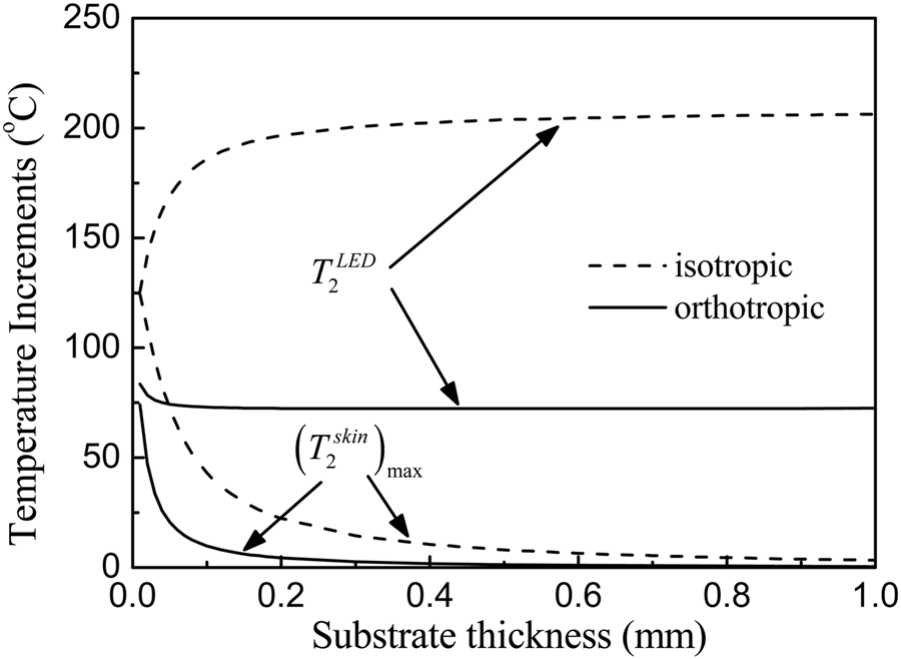
Effects of the substrate thickness on the μ-ILED temperature increase and the maximum skin temperature increase under a constant power of 10 mW.

The substrate thickness has a significant influence on the temperature increase. Figure 4 shows the influences of the substrate thickness on the μ-ILED temperature increase ${T}_{2}^{LED}$ and the maximum temperature increase ${({T}_{2}^{skin})}_{\max }$ in the skin under a constant power of 10 mW. As the substrate thickness increases, both ${T}_{2}^{LED}$ and ${({T}_{2}^{skin})}_{\max }$ decreases sharply first and then reaches to be steady values slowly. Comparing to the case with the use of isotropic substrate (dash line in Fig. 4), the orthotropic substrate can decrease both ${T}_{2}^{LED}$ and ${({T}_{2}^{skin})}_{\max }$ significantly even for a very thin thickness. For example, ${T}_{2}^{LED}$ drops 56.2% from 169.7 °C to 74.4 °C and ${({T}_{2}^{skin})}_{\max }$ drops 71.5% from 72.3 °C to 20.6 °C for a thin thickness of 50 μm for ${k}_{sub}^{x}/{k}_{sub}^{z}=10$. These results can help the design of μ-ILEDs for biointegrated applications under a constant power.

**Figure 5:**
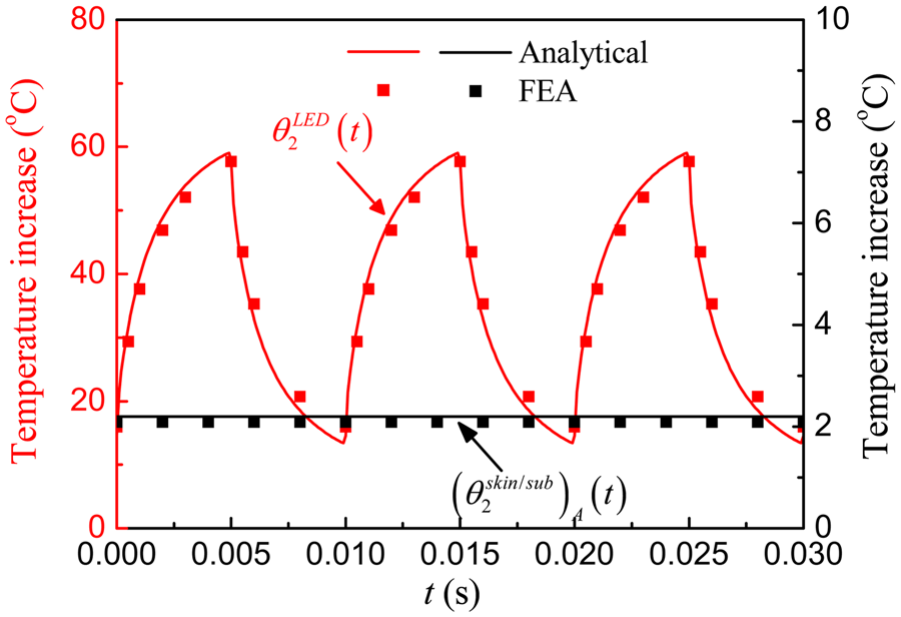
The μ-ILED temperature increase and the temperature increase at point *A* on the skin/substrate interface versus time under a pulsed power with the duty cycle 50% and period 10 ms for the thickness of substrate of 200 μm.

**Figure 6:**
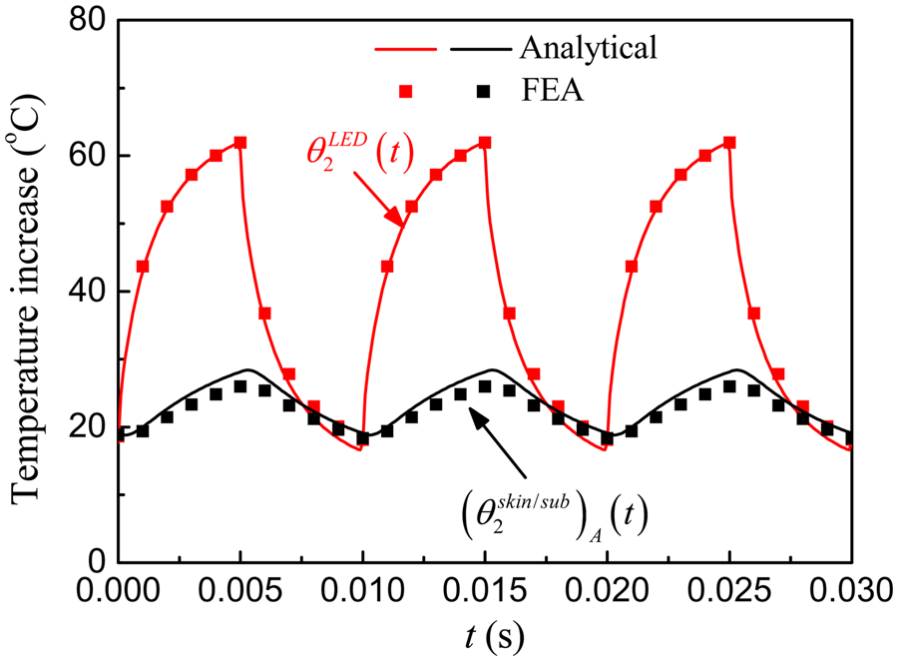
The μ-ILED temperature increase and the temperature increase at point *A* on the skin/substrate interface versus time under a pulsed power with the duty cycle 50% and period 10 ms for the thickness of substrate of 20 μm.

Under a pulsed power, the temperature after saturation will oscillate within a constant band. Figure 5 shows the μ-ILED temperature increase ${\theta }_{2}^{LED}(t)$ and the temperature increase ${({\theta }_{2}^{skin/sub})}_{A}(t)$ at point *A* (0, 0, *h*_*encap*_ + *h*_*sub*_) on the skin/substrate interface versus the time under a pulsed power with the duty cycle 50% and period 10 ms. The analytical predications agree well with FEA. The μ-ILED temperature increase ${\theta }_{2}^{LED}(t)$ after saturation oscillates from 13.5 °C to 59.0 °C. The temperature increase ${({\theta }_{2}^{skin/sub})}_{A}(t)$ remains a constant because the substrate thickness is set as 200 μm, which is large enough to insulate the heat oscillation from the μ-ILED heat source. For a thinner substrate with the thickness of 20 μm as shown in Fig. 6, the temperature increase ${({\theta }_{2}^{skin/sub})}_{A}(t)$ is no longer a constant and also oscillates since more heat will transfer into the human skin.

**Figure 7:**
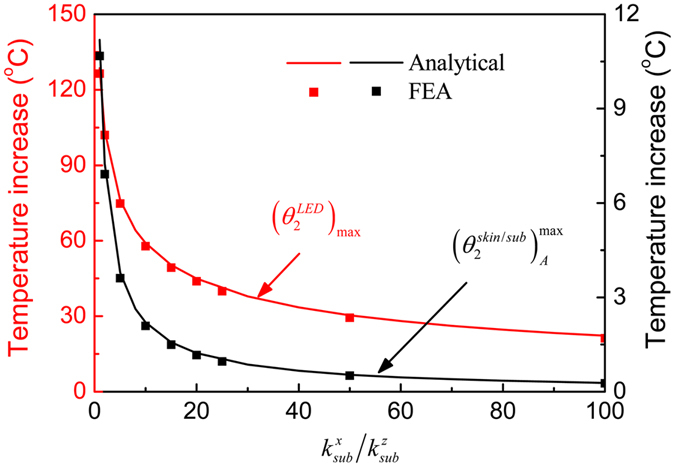
Effects of the orthotropic substrate on the maximum μ-ILED temperature increase and the maximum skin temperature increase under a pulsed power with the duty cycle 50% and period 10 ms.

**Figure 8:**
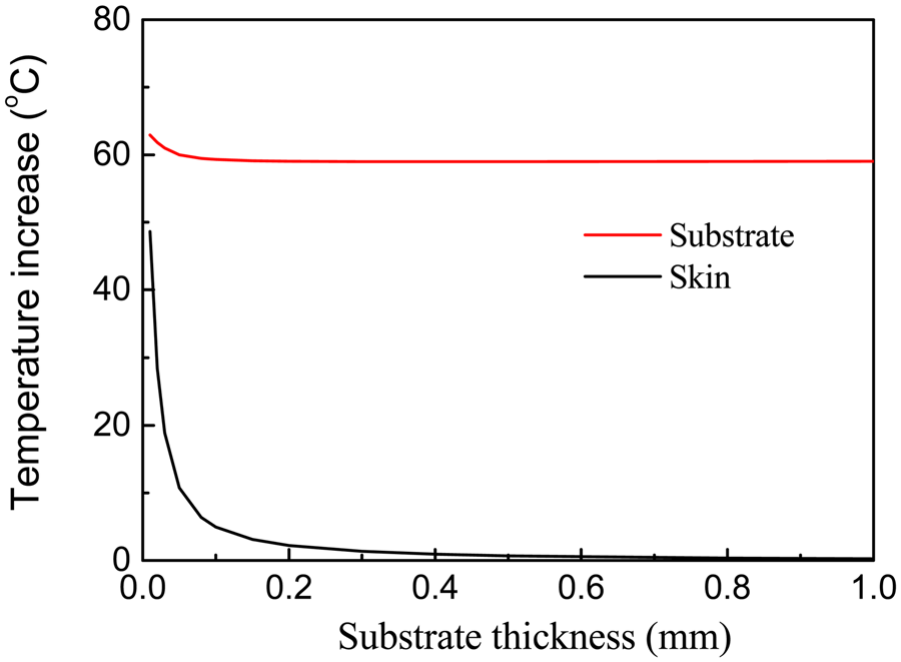
Effects of the substrate thickness on the μ-ILED temperature increase and the maximum skin temperature increase under a pulsed power with the duty cycle 50% and period 10 ms.

Figure 7 shows the influences of the orthotropic substrate on the maximum μ-ILED temperature increase ${({\theta }_{2}^{LED})}_{\max }$ and the maximum temperature increase ${({\theta }_{2}^{skin/sub})}_{A}^{\max }$ in the skin. The thermal conductivity of the substrate along the *z* direction is fixed as ${k}_{sub}^{z}=0.17\,W/m/K$. As the thermal conductivity ratio increases from 1, corresponding to an isotropic substrate, to 30, temperature increase ${({\theta }_{2}^{LED})}_{\max }$ drops 70.4% from 128.6 °C to 38.0 °C and the maximum temperature increase ${({\theta }_{2}^{skin/sub})}_{A}^{\max }$ drops 92.3% from 11.2 °C to 0.86 °C, and both of them drop slowly as the thermal conductivity ratio further increases. This trend is very similar to that for the case of constant power, as shown in Fig. 3. Figure 8 shows the influences of substrate thickness on the maximum temperature increase of μ-ILED and maximum temperature increase in human skin under a pulsed power with the duty cycle 50% and period 10 ms. The results show that with the increase of substrate thickness, the maximum temperature increase of μ-ILED and maximum temperature increase in human skin both decrease to a constant. The maximum temperature increase of μ-ILED drops from 62.9 °C to 59.0 °C, and the maximum temperature increase in human skin drops from 48.6 °C to 0.2 °C with substrate thickness increasing from 0.01 mm to 1 mm.

**Figure 9:**
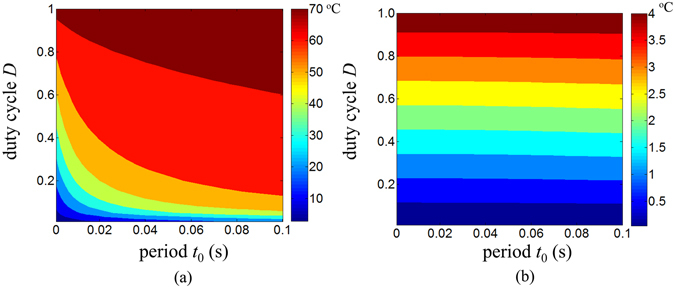
The effects of duty cycle and period on (**a**) the maximum μ-ILED temperature increase and (**b**) the maximum skin temperature increase under a pulsed power.

Figure 9 shows the influences of duty cycle and period on the maximum μ-ILED temperature increase ${({\theta }_{2}^{LED})}_{\max }$ and the maximum temperature increase ${({\theta }_{2}^{skin/sub})}_{A}^{\max }$ in the skin in a pulsed operation. The reductions of the duty cycle and period (<0.1 s) are helpful to decrease the maximum μ-ILED temperature increase as shown in Fig. 9a. From Fig. 9b, the maximum temperature increase ${({\theta }_{2}^{skin/sub})}_{A}^{\max }$ in the skin decreases almost linearly with the decrease of the duty cycle but it is almost independent on the period due to the thick substrate (200 μm) used in the calculations.

## Conclusions

In summary, three-dimensional analytical models, validated by FEA, are established to investigate the thermal behaviors of μ-ILEDs on an orthotropic substrate integrated with human skin. Both the operations of μ-ILEDs in a constant mode and pulsed mode are studied. The coupling between the Fourier heat conduction in the μ-ILED system and the Pennes bioheat transfer in the human skin is accounted in the model. It is shown that the orthotropic substrate, which can control the heat flow directions, can help to reduce the maximum μ-ILED and tissue temperature increase simultaneously and shows unique benefits in thermal management. The influences of thermal conductivities of the orthotropic substrate, substrate thickness, and loading parameters (e.g., duty cycle, pulse period) on the maximum μ-ILED and tissue temperature increase are investigated. These analytical models can be easily extended to study μ-ILED system with different layouts and materials for various biointegrated applications.
